# Describing Sources of Uncertainty in Cancer Drug Formulary Priority Setting across Canada

**DOI:** 10.3390/curroncol28040236

**Published:** 2021-07-17

**Authors:** Kristina Jenei, Stuart Peacock, Michael Burgess, Craig Mitton

**Affiliations:** 1 School of Population and Public Health, University of British Columbia, Vancouver, BC V6T 1Z3, Canada; michael.burgess@ubc.ca (M.B.); craig.mitton@ubc.ca (C.M.); 2 Canadian Control Research, BC Cancer, Canadian Centre for Applied Research in Cancer Control (ARCC), Vancouver, BC V5Z 4E6, Canada; speacock@bccrc.ca; 3 Faculty of Health Sciences, Simon Fraser University, Burnaby, BC V5A 1S6, Canada; 4 W. Maurice Young Centre for Applied Ethics, University of British Columbia, Vancouver, BC V6T 1Z3, Canada

**Keywords:** health technology assessment, oncology, cancer drugs, uncertainty, qualitative interviews, reimbursement recommendations

## Abstract

Over the years, there have been significant advances in oncology. However, the rate that therapeutics come to market has increased, while the strength of evidence has decreased. Currently, there is limited understanding about how these uncertainties are managed in provincial funding decisions for cancer therapeutics. We conducted qualitative interviews with six senior officials from four different Canadian provinces (British Columbia, Alberta, Quebec, and Ontario) and a document review of the uncertainties found in submissions to the pan-Canadian Oncology Drug Review (pCODR). Participants reported considerable uncertainty related to a lack of solid clinical evidence (early-phase clinical trials: generalizability, immature data, and the use of unvalidated surrogate outcomes). Proposed strategies to deal with the uncertainty included risk-sharing agreements, collection of real-world evidence (RWE), and ongoing collaboration between federal groups and provinces. The document review added to the reported uncertainties by classifying them into five main categories: trial validity, population, comparators, outcomes, and intervention. This study highlights how decision makers must deal with significant amounts of uncertainty in funding decisions for cancer drugs, most of which stems from methodological limitations in clinical trials. There is a critical need for transparent priority-setting processes and mechanisms to reevaluate drugs to ensure benefit given the high level of uncertainty of novel therapeutics.

## 1. Introduction

Decision making for funding cancer drugs has become increasingly complex in recent years. This is partly due to a rise in cancer incidence, survivorship, drug costs, and subsequent health system expenditure. More recently, these factors have been coupled with the concern that many novel therapies are associated with considerable uncertainty due to methodological limitations from clinical trials. Uncertainty from clinical studies flows into economic evaluations and introduces additional challenges in formulary decision making. While economic evaluations can incorporate uncertainty into models using various methodological techniques, the rapidly increasing numbers of experimental and costly drugs in oncology adds new complexities that challenge traditional methods [1]. Then, these evaluations must be made on several assumptions related to long-term effectiveness, adoption feasibilities, and quality of life.

Any uncertainty about the magnitude of benefit of new therapeutics from clinical trial evidence and subsequent economic evaluations can be exacerbated by “external factors” such as political priorities and pressure from media, industry, patients, and clinicians [2]. For example, one Canadian study demonstrated that media coverage had significant effects on generating positive and faster funding decisions when compared to other drugs with similar benefit that received less media coverage [3]. Furthermore, there is a concern about financial conflicts between clinicians (and, at times, patient representatives) and the pharmaceutical industry. In Canada, 66.3% of all submissions to pan-Canadian Oncology Drug Review (pCODR) reported some kind of financial conflict [4]. Half of these were directly with the drug under review. Furthermore, a study from the United States demonstrated that financial conflicts of interest might also extend to patient representatives and advocacy groups [5]. These challenges, among others, translate into a growing skepticism for the reported benefit of new cancer drugs [6,7].

For a drug to come to market in Canada, it must be approved by Health Canada, evaluated by the pCODR (or the Institute National d’Excellence en Sante (INESSS) in Quebec), and funded for reimbursement at the provincial level. While Health Canada, the Canadian Agency for Drugs and Technology in Health (CADTH), and INESSS review similar evidence, their goals vary slightly. Health Canada focuses primarily on safety and efficacy, whereas CADTH and INESSS evaluate therapeutics for value based on specified criteria. Health care financing and delivery is primarily the responsibility of provincial governments. For cancer care, provinces generally rely on the recommendations from the pCODR program at CADTH. pCODR assesses the value of a new therapeutics through a deliberative framework that includes clinical and economic evidence, patient input, and adoption feasibility. The submissions are evaluated by pCODR and then reviewed by the pCODR Expert Review Committee (pERC). pERC is comprised mainly of medical oncologists; however, it does include other membership such as pharmacists, patient representatives, health economists, an ethicist, and non-oncologist physicians. Once a submission is reviewed, pERC provides provincial and territorial decision makers with recommendations regarding whether a drug should be reimbursed. Some provinces have also established their own review boards such as the Priorities Evaluation Committee in BC and the Ontario Steering Committee on Cancer Drugs in ON [8].

Despite pCODR support in reimbursement recommendations, it is known that resource allocation occurs differently across provinces. Public coverage for the same drug can differ province to province, which has led to concern about possible “fragmentation” of the cancer system across Canadian provinces [8]. Dependent on the jurisdiction, one drug might be covered under a public plan, whereas it will not be covered in another. For example, oral cancer drugs receive universal coverage in Saskatchewan. This differs from Newfoundland, where patients must qualify for provincial assistance to access the same drugs. This variation can lead to significant financial implications for patients and raise ethical concerns about differential access within the same country [9].

As oncology enters into an era of precision medicine—where evidence becomes more complex—decision makers are increasingly forced to make reimbursement decisions on the basis of uncertain evidence that can have significant opportunity costs [10]. Despite recent research to determine methodological challenges in clinical trials and economic evaluation, there is little knowledge as to what uncertainties provincial decision makers encounter in cancer drug reimbursement decisions. Therefore, the objective of this study was to explore how uncertainty is understood and managed in provincial funding decisions for cancer drugs across Canada. To do this, a two-phased approach was utilized: key informant interviews with provincial decision makers and a document review for the uncertainties identified in pCODR review documents.

## 2. Materials and Methods

### 2.1. Study Design

A semi-structured interview guide was developed to assess funding processes, the nature and weight of various sources of evidence (clinical, economic, public, or patient input), and the role of federal bodies such as pCODR and the pCPA with key informants. Respondents shared characteristics of being either a ‘decision-maker’ or someone “involved in decisions for funding cancer drugs” and were purposively sampled from their jurisdiction. All interviews were audio-recorded with permission and transcribed verbatim.

To supplement the key informant interviews, submissions to pCODR between 1 January 2015 and 31 December 2019 were reviewed for uncertainties. Uncertainty meant “an unresolved issue, limitation or methodological deficiency”. This definition has been used in prior studies [11]. The search was restricted to completed evaluations for solid tumors that received a final funding recommendation. Drugs that were reviewed for multiple tumor types were included as distinct entries for the analysis. One report per tumor type was included in this analysis to ensure a manageable qualitative sample. This type of inclusion and exclusion criteria has been applied in prior studies that have analyzed pCODR submission documents [12]. Any inconsistencies in the pERC documents were resolved with further analysis of the Final Clinical Guidance Report. Submission data were publicly available online; therefore, no institutional ethics approval was required.

### 2.2. Analysis

Qualitative content analysis was used to analyze the interviews and pCODR documents. Based on prior knowledge from the literature review and key informant interviews, a data extraction table was constructed a priori. Following qualitative descriptive methodology, codes were reviewed and compiled in broad categories [13]. NVivo qualitative software 12.6.0 was used to organize themes. Each review comment that indicated a form of uncertainty was counted once. For example, if there were multiple indications of bias mentioned in the submission document, one point would be counted in the “trial validity” category. Quantitative content analysis was used to indicate the frequency at which the categories occurred.

## 3. Results

### 3.1. Sources of Uncertainty Identified in Key Informant Interviews

Overall, a total of six key informant interviews were included from provincial cancer agencies (2), ministries of health (1), and national or provincial HTA organizations (3). Participants were from four provinces: BC (1), AB (1), ON (1), and QC (2) and held a variety of roles including senior management, directors of oncology programs, health technology assessment (HTA) methodologists, and members of drug review or advisory committees.

The key informant interviews identified four main sources of uncertainty in funding decisions for cancer therapeutics: (1) a lack of a solid evidence base due to methodological limitations from clinical trials, (2) increasing costs, (3) external influences such as pressure from the FDA, patients and clinicians, and political will, and (4) strategies to manage uncertainty. These themes, along with illustrative quotations, are summarized in Table 1.

#### 3.1.1. Uncertainty about the Clinical Evidence

The most common source of uncertainty identified was uncertainty about the clinical evidence. Participants discussed challenges in the context of clinical trials, which has historically been used as the foundation for the risk benefit threshold. Subthemes included concerns about selective recruitment based on favorable performance status that make it difficult to generalize the effectiveness of a therapeutic to the provincial population. Trial participants are often heavily treated or selectively enrolled. Furthermore, once a drug is approved for a certain demographic, there is considerable pressure to expand its use to patients who have shown poor responses to other drugs, in which the evidence is more uncertain. Participants noted increased uncertainty when new therapeutics do not demonstrate improvements in patient-centered outcomes such as overall survival and quality of life. Furthermore, it is hard to fund a drug that has not been compared to the current standard of care. The concerns related to clinical evidence were exacerbated by the increasing pace of drug approvals at the federal level, which translates into a faster flow from federal bodies to provincial decision makers.

#### 3.1.2. Uncertainty about Drug Costs

Every participant acknowledged the rising cost of oncology drugs as a major source of uncertainty; however, decision makers were hesitant to acknowledge that this factor alone would affect the outcome of the funding decision. Two participants from QC explicitly stated that efficacy is the first criteria for a drug funding recommendation, even if it means funding a drug based on small incremental benefits and significantly higher costs. Furthermore, all participants noted that the budget impact is often underestimated, especially with new targeted therapies, which add significant burdens in their adoption in health care systems.

#### 3.1.3. Uncertainty from ‘External Factors’

Participants noted several challenges from outside of the regulatory processes. These included patient and clinician pressure, US Food and Drug Administration (FDA), industry, media, and political influences. Participants noted additional uncertainty from clinician and patient advocacy groups. Understandably, these groups have different goals from decision makers. Patients and clinicians focus on individual need rather than the population and therefore might have higher tolerance for uncertainty. Furthermore, a clinician participant noted that the proximity of the United States to Canada creates pressure for payers to make similar funding decisions and adds challenges to implement standards for value. Other sources of uncertainty included media and industry, which can influence the public and political members to adopt certain therapeutics contrary to the evidence. Participants often expressed ambiguity about the power of political agendas.

#### 3.1.4. Strategies to Manage Uncertainty

Participants suggested various approaches to managing uncertainty in provincial drug funding decisions. These strategies fell into four categories: financial risk management, real world evidence, reassessment of drugs with uncertain benefit, and the opportunity to participate in pan-Canadian collaborations. Participants discussed risk-sharing agreements between public payers and industry to manage uncertainty. Payers seemed to favor sharing the risks and cost burden with the pharmaceutical companies, especially in the initial stages of adoption where uncertainty is at its highest. All participants seemed to agree on the importance of collecting RWE and, at times, even negotiating these conditions into the initial contracts with pharmaceutical companies. However, challenges noted for collecting RWE stem from a historical reliance on clinical trials in oncology and subsequent reluctance to use other methods such as observational designs. All participants noted that reassessment of the treatment space generally does not occur and that this is a current gap. There were numerous reasons for this, including the regulatory approval pace, limited human and financial resources to reassess drugs, political will, and a lack of systematic ways for disinvestment.

Pan-Canadian collaborations such as pCODR, pCPA, CDIAC, and others have increased opportunities for collaboration, partnership, and transparency and had positive impacts on managing uncertainty in funding decisions. Positive aspects included streamlined processes based on one federal HTA process that generates comprehensive reports that identify areas of uncertainty. Furthermore, another positive was “less neighbor checking” between provinces. One decision maker noted that prior to the formation of pCODR, some provincial payers would monitor what drugs other provinces were funding and make decisions based on this. However, with one recommendation from pCODR, provinces can rely on one source of reliable evidence to make their own decisions. However, one participant noted that pCODR might also contribute to uncertainty in formulary decisions as there is no obvious bar for what constitutes as “good evidence.” This can incentivize manufacturers to submit earlier evidence, as there seems to be little consistency for what receives a positive or negative recommendation in terms of immature evidence. However, other decision makers noted that it was not clear who ought to be setting a bar for the standard of evidence—whether that be Health Canada or CADTH. Payers are struggling to understand what is acceptable.

### 3.2. Sources of Uncertainty in pCODR Submissions 

Overall, there were a total of 73 distinct submissions to pCODR between 2015 and 2019. One report per tumor type was included in this analysis, for a total of 47 distinct submissions included in the qualitative analysis. The pCODR document review identified five sources of uncertainty in the clinical evidence. These included issues with trial validity, non-generalizable population, lack of or unreliable comparators, unvalidated or missing outcome measurements, and resource intensive implementation. An overview of categories along with representative quotations can be found in Table 2.

#### 3.2.1. Trial Validity

In approximately 50% of the submissions, reviewers noted uncertainties related to methodological limitations in the study design, which raised questions about meaningful long-term benefit. The most common were selection bias, reporting bias, performance bias, and attrition bias. Selection bias can occur when patients are selectively recruited into trials based on characteristics that differ from the wider population. For example, some participants were chosen on performance status, which might ensure resilience to withstand treatment and can lead to better outcomes. Furthermore, reporting and performance bias can be introduced when patients and clinicians are aware of the treatment assignments. Uncertainty related to attrition bias is introduced when participants exit studies for unknown reasons. One common example was data related to health-related quality of life where data are collected from individuals who remain in the study rather than all initial study participants.

#### 3.2.2. Study Population

In 47% of the submissions, there was uncertainty about whether the sample of participants in clinical trials is generalizable to clinical practice. Due to the investigation of often rare molecular alterations, it can be difficult for clinical trials to recruit enough participants to adequately power a trial. To remedy this, trials can have an international scope where patients are enrolled from centers around the world. The varying demographics from those enrolled can create uncertainty about its effectiveness in a specific population, such as Canada. Indeed, assessing the generalizability of the trial results can be a challenge for all countries in all international trials 

#### 3.2.3. Study Comparators

In 40% of the submissions, reviewers noted how the lack or inappropriate use of a comparator added uncertainty. It is common that trials do not compare to clinically relevant standards of care. At times, trials will only have an experiment arm or compare against placebo. This can make a drug benefit appear substantial. However, this might not be the case. It can be hard to make a reimbursement recommendation when there is uncertainty to its performance compared to the drug used in current clinical practice.

#### 3.2.4. Study Outcomes

Uncertainties related to outcomes were indicated in 72% of the submissions and fell into two categories: the use of unvalidated endpoints and missing data related to important patient-centered outcomes such as overall survival and health-related quality of life. There were numerous issues with the use of unvalidated endpoints for certain tumor types, which raised questions about whether the efficacy translated into effectiveness in real world conditions. Furthermore, there were additional uncertainties when trials did not collect important patient-centered information such as health-related quality of life.

#### 3.2.5. Interventions

Although this analysis focused on clinical uncertainties, there were substantial references to the resources required for implementation of a therapeutic. These uncertainties were indicated in almost all of the submissions (83%) and were related to the duration of treatment, adoption feasibilities, and administration of the drug. The duration of a treatment has important effects of the quality of life of a patient. There were additional uncertainties related to the administration of certain therapeutics. Some therapeutics require additional resources such as staff with special training or new facilities for administration. The uncertainties generated from the duration of treatment and resource-intensive administration flow into considerations for their adoption in a health care system. It is often the case that the therapeutic itself is also costly. Therefore, high costs and additional resources creates uncertainty for the feasibility of its adoption.

## 4. Discussion

The key informant interviews confirm that there are many areas of uncertainty that challenge decision makers in oncology, most of which relate to evidence from clinical trials. The results from the pCODR document review add to this finding by systematically categorizing these uncertainties into five main themes. Both sets of findings demonstrate how uncertainty is prevalent in oncology. Overall, pCODR and payers raised similar concerns, especially about the generalizability of the trial results into a broader patient population. However, payers were more concerned about how a therapeutic will translate into existing lines of treatment, whereas pCODR focused on recommendations for one submission at a time.

Provinces generally rely on the reimbursement recommendations from pCODR. However, the document review revealed many sources of uncertainty that are embedded within these evaluations. These findings are not surprising, given the quality of evidence submitted to pCODR by manufacturers. For example, Meyers et al. (2021) found that over half (53.9%) of positive recommendations by pCODR between 2011 and 2020 were based on a surrogate endpoint, such as PFS, while only a minority offered OS gains (32.1%). Of the drugs that demonstrated OS benefit, the median survival gain was only 3.7 months [14]. An analysis of drugs approved by the European Medicines Agency demonstrated similar consequences to relying on surrogate measures: a median OS benefit of 2.7 months [15]. Furthermore, Raymakers et al. (2021) found that nearly a quarter (24%) of submissions to pCODR were made on the basis of early phase clinical trials (Phase I or II) [16]. Other pCODR analyses have demonstrated that the majority of submissions between 2015 and 2018 lacked any data on quality of life endpoints [12].

Drugs submitted with suboptimal evidence from clinical trials exacerbate regulatory weaknesses. For example, uncertain therapeutics often receive conditional recommendations from pCODR. However, it has been shown that provinces often interpret conditional recommendations as positive endorsements [17]. This can be problematic as therapeutics that receive conditional recommendations are rarely revisited [10]. Raymakers et al. provide a specific example of olaratumab (for soft tissue sarcoma), which remains conditionally listed by pCODR despite subsequent studies not confirming the reported benefits from the pivotal Phase II trial [16]. Decisions based on uncertain data without a plan for reassessment can introduce additional risks and opportunity costs for patients.

If trials with immature data continue to be used for reimbursement decisions, analytical methods and mechanisms for reassessment require strengthening [16].

Participants offered suggestions for ways uncertainty can be managed in oncology. These strategies included the collection of RWE, outcome-based agreements, and mechanisms for reassessment. Many participants discussed risk-sharing agreements between public payers and industry to manage uncertainty. There are some innovative strategies internationally. For example, one participant cited a Cancer Drugs Fund from NICE [18] (in the United Kingdom) that is utilized to grant patient access to promising but uncertain therapeutics while additional RWE evidence can be collected. Other participants were in favor of outcome-based agreements where provincial funders pay manufacturers based on positive patient outcomes from the therapeutic. This way, the uncertainty is shared. All participants seemed to agree on the importance of collecting RWE and even negotiating conditions into contracts with manufacturer. Furthermore, the key informants noted the absence of mechanisms to revisit drugs with uncertain benefit despite their interest in the ways this might occur, which is otherwise known as “health technology management” [19]. Challenges for these strategies include limited resources and pace. Participants stated that reassessment was a resource-intensive process, especially in provinces with segregated health care systems. Furthermore, the pace from approval to payer is increasing, and participants often noted how by the time they collected evidence for the most recent drug, the next one would arrive. Future research might assess the resources necessary to implement these strategies.

Given the uncertainty of benefit with new therapeutics and potential for external factors to affect the decision-making process, there is a need for more transparent frameworks at all levels of the regulatory and reimbursement process (e.g., Health Canada, pCODR, INESSS, and provinces). The key informants all noted that they could name the pieces of the evidence in the provincial decision-making process but did not know exactly how each one was weighed in the final decision. pCODR publishes a deliberative framework that includes addressing four criteria: clinical, economic, patient values, and adoption feasibility. However, the weighting scheme of each of these components is unknown. It might be assumed that each review team apply their own implicit weights to these criteria, which create questions about the consistency of the process [20]. A recent empirical analysis of pCODR recommendations determined that clinical evidence, such as efficacy, appear to carry the greatest weight whereas cost-effectiveness did not seem to have any effect at all [21]. Similarly, all key informants noted how costs did not play a significant role in the funding decision, and two decision makers from QC specifically described how they would fund a drug based on small incremental benefit even if it was significantly more expensive. The justification for this was to provide transparency to the funding process with an intentional weight placed on efficacy as the primary criterion for adoption. They noted how other HTA bodies have multiple criterions, for example “cost-effectiveness” and “patient values”, but it is unknown how much weight each criterion was given in the final decision. A larger weight on efficacy was meant to add consistency to formulary decisions. These findings are surprising given the objective to assess the value of new medicines. The cost-effectiveness of a given therapeutic is integral to the formulary decision given constrained budgets in publicly funded health care systems. Furthermore, economic analyses (such as cost-effectiveness) provide important information for how to maximize patient outcomes with given resources while comparing new therapeutics with current standards of care. This is especially important given that pharmaceutical manufacturers often submit evidence from clinical trials with inappropriate or irrelevant control arms that do not demonstrate superiority over what is used in clinical settings [22].

The lack of formal priority-setting processes for implementing and reassessing therapeutics has created a challenging environment for Canadian decision makers in cancer control. This challenge, combined with the high levels of uncertainty from pivotal clinical trials identified during the document review, means that making decisions with limited evidence is a reality for those involved in provincial cancer drug funding. However, it is also evident from this study that there is an appetite for mechanisms to manage uncertainty, namely ways for ongoing reassessment of uncertain therapeutics. With the rise of complex study designs (e.g., basket trials) and methodological limitations in clinical trials (e.g., unvalidated surrogate endpoints), there is a question as to which agency (Health Canada, CADTH, INESSS, or provinces) ought to set the bar for quality as payers are struggling to understand what is acceptable evidence for reimbursement. Without some evidentiary standard, manufacturers will continue to submit suboptimal evidence. Future policy initiatives and research ought to investigate how health technology management might occur in the Canadian institutional setting. Furthermore, mechanisms to strengthen follow-up for conditional recommendations and RWE collection are needed.

### Limitations

The current study provides a qualitative analysis of the uncertainties for oncology drugs at the time of the provincial reimbursement decision in Canada. However, the results should be interpreted in light of certain methodologic limitations. First, the key informant interviews were from a small group of participants; therefore, it might be hard to generalize the findings to all settings. However, concerns related to a small sample size are not as limiting in qualitative analyses. In qualitative designs, a small number of interviews can be analyzed with more depth, increasing the internal validity of the study [13]. Furthermore, interviews with senior decision makers who are accountable to their organizations and the public can stimulate political responses. This means that some questions were answered in ways that might not be representative of what happens in practice. To offset this concern, recruitment ensured a diversity of participants involved in various aspects of the decision-making process.

The pCODR document review also should be interpreted in light of other limitations. First, the study relied on publicly available documents. Since pCODR considers all available evidence in its evaluation process, some of which may not be clear in public documents, it might be difficult to draw conclusions solely examining one portion of the process. Furthermore, the document review was limited to comments of the pCODR reviewer in lieu of an independent critical appraisal. This is important to consider, as a critical appraisal would likely generate a more realistic and perhaps more substantial list of uncertainties. This is especially relevant in the quantitative content analysis to indicate the frequency at which these uncertainties occurred. However, similar studies have found comparable results. For example, Naci et al. (2019) used the Cochrane risk of bias tool to critically appraise the trials of the drugs approved by the European Medicines Agency [23]. The study found that nearly half of the clinical trials were assessed to be high risk of bias due to limitations to study design and analysis. Similarly, this study found that approximately 50% had issues related to trial validity. Another limitation is the choice in the exclusion and inclusion criteria applied per drug submission. This resulted in a total of 47 out of 74 submissions that were included in the qualitative analysis. It is recognized that this type of criteria can introduce selection bias to the study. However, after the criteria were applied, the characteristics of the drugs included in this analysis were comparable to characteristics of those found in a database of all submissions to pCODR since inception.

## 5. Conclusions

The key informant interviews identified numerous sources of uncertainty, many of which were associated with a lack of solid clinical evidence, which stemmed from methodological limitations in clinical trials. The pCODR document review adds to these findings by systematically categorizing uncertainties into five main categories. These categories highlight the substantial challenges decision makers face when funding therapeutics at a provincial level. Many of the uncertainties in the document review were raised by provincial decision makers. However, one major difference was the focus on the management of a therapeutic space in contrast to pCODR, which generally focuses on individual drug submissions. Although there has been federal movement to focus on implementation with the incorporation of agencies such as CDIAC into the pCODR process, the impact has yet to be evaluated. This study raises important questions about the evidential standard in oncology and how might this adapt in the era of precision medicine.

## Figures and Tables

**Table 1:** Illustrative quotes for themes of uncertainty identified by Canadian decision makers.

THEMES	QUOTES
**CLINICAL EVIDENCE**	
SELECTIVE RECRUITMENT	“It’s the patient population, the previous treatment, make that uncertainty.”—Participant 6 (Clinician) “...A common [source of uncertainty] is good performance status patient. Most clinical trials restrict patients to good performance status. But there is considerable pressure then once you’ve got the drug, particularly if they don’t have too many side effects, is to just expand the population and use it with patients with poor performance status. We just don’t know whether it’s going to be beneficial in that situation. But there is considerable pressure to fund it.”—Participant 1 (Senior Executive)
SURROGATE ENDPOINTS	“I think [it’s] the clinical evidence and then just more and more pressure to fund drugs based on more limited or limited evidence. So randomized Phase 2′s, response rates from phase 1 [trials] and more.”—Participant 6 (Clinician) “We are trying—as payers—to buy better patient outcomes.”—Participant 2 (Senior Executive)
PACE	“There are some therapeutic spaces, some cancer types, that change so quickly that before you know it, the drug is only funded for a couple of years, and then the next thing comes along. Then, it is a different landscape altogether. There are some spaces that it does not change as much.”—Participant 6 (Clinician) “But the challenge with drugs, particularly cancer drugs (or maybe any drugs), is that things move so fast. And our ability to tolerate the historical, ‘OK, it’s going to take us four or five years to actually get an answer.’ Things will have moved on and there’s new drugs and you’ve invested all of these resources to see whether something is behaving how you thought it would in the real world. And there’s three new drugs in that cancer treatment space. And nobody cares.”—Participant 1 (Senior Executive)
**DRUG COSTS**	
HESITANCY TO WEIGHT COSTS	“We take into account the efficacy as number one and then look at other criteria, whereas other HTA bodies will amalgamate multiple different criteria, including the economic cost considerations with efficacy. So, you have that weight on both of them, which I thought was really interesting about our process because we seem very explicit in the therapeutic value is number one.”—Participant 3 (Senior Executive)
BUDGET IMPACT	“So, the budget impact is so huge on that because I mean, what I’ve read with CAR-T is that it’s not just the $450,000 infusion or process, but it’s also the side effects. It’s the rooms that you need, the trained clinicians, the hospital, long-term hospitalization.”—Participant 2 (Senior Executive)
**EXTERNAL INFLUENCES**	
PATIENT OR CLINICIAN PRESSURE	“I guess the challenge always is from a clinician perspective. They don’t always consider the cost. In fact, they recognize that these drugs are costly, but it doesn’t seem to slow down or reduce the pressure to fund them. […] In the face of a cancer diagnosis and the treatment that potentially could help, cost is not something that they want to take into consideration.”—Participant 1 (Senior Executive)
US FOOD AND DRUG ADMINISTRATION	“So, their [FDA] bar for approval is low. They don’t have to think about the price, although people do have to think about the price. So, the bar is “it has some signal of activity and it doesn’t immediately kill people”. Then, that drives the demand for drugs that potentially may help somebody in a situation when maybe they don’t have great choices or a cancer that would actually kill them. So that drives the clinical demand for us here in Canada. So, then that makes it very difficult for us to then impose an additional bar around what value is it providing and what prices or the cost effectiveness is in a culture that wants to use drugs whenever they want to use them.”—Participant 6 (Clinician)
INDUSTRY “HYPE”	“They [industry] thought it was going to be a cure, well, I’m hearing them temper it down. ‘Well, you might get a few years.’ That’s not a cure!”—Participant 2 (Senior Executive)
POLITICAL INFLUENCES	“We spent a summer going through all the drugs with the tumor groups saying, ‘Okay, what could we de-list if you want to free up money for these newer drugs.’ So, we went to our board with actually what I considered were underperforming drugs. And they said, ‘No, no, no. We’ll find the money.’ And I went, ‘Really??’ You know, so I don’t ever underestimate the politicalness of this stuff.”—Participant 3 (Senior Executive)
**MANAGING UNCERTAINTY**	
FINANCIAL RISK MANAGEMENT	“And if it [the new drug] is iffy or is uncertain, they are throwing the drug into the cancer drug fund middle space where there is shared funding while they develop the real-world evidence to feed into NICE so that they can say yes or no. It’s a two-year probation space. And then it’s not de-listing or listing too early. It’s a shared space where the funding isn’t at the payer level or the industry—it’s shared.”—Participant 2 (Senior Executive) “I just think I was the only thing that I noticed prior to negotiating nationally, we had done some pretty creative stuff I thought. We did pay for performance where the pivotal trial expected this survival. And it was like maybe fifteen patients a year. So, we actually entered into a contract with a manufacturer that [stipulated] we were going to pay for performance. So, we got different rebates depending on our patient’s survival. But it worked really well, and we tried it out.”—Participant 3 (Senior Executive)
REAL WORLD EVIDENCE	“If we were to collect data to find out their true experience from a payer’s perspective, and not just clinical trial data that’s based on a highly selected group that happens to be healthy enough to be in the trial.”—Participant 4 (Senior Executive) “Part of that, again, just stems from the fact that they know, and they trust something that’s called a trial even if there is no actual randomization or even if there’s no actual control arm—you know, it’s a trial.”—Participant 5 (HTA Methodologist)
REASSESSMENT FOR DRUGS WITH UNCERTAIN BENEFIT	“We don’t [reassess drugs]—not in the formalized way that we list drugs. So that is currently a flaw. A part of it is there is so much pressure to list drugs that it’s difficult to use the limited resources you have in order to make the listing of drugs work to apply to de-listing. And as you know, it’s difficult to de-list once something is accepted and people are using it.”—Participant 1 (Senior Executive)
PAN-CANADIAN COLLABORATIONS	So even though we talk about provincial, [there are] different processes and parallel patchwork processes; before, [it] was worse. It’s actually better now in my opinion.”—Participant 6 (Clinician) “I think the pan-Canadian oncology review is getting, and has been really good, at calling out uncertainty. But do they [pCODR] contribute to uncertainty? Absolutely. Because they keep moving the bar. So, you know a company can’t predict what’s coming out of pCODR. They should be able to predict and therefore not put stuff in when it’s too early in evidence. But they are throwing it at it [pCODR] because sometimes they let it through and sometimes, they don’t.”—Participant 2 (Senior Executive) “Oh, they [pCODR] definitely help. The reviews that CADTH does are very helpful, it is very good, and thorough.”—Participant 1 (Senior Executive)

CADTH = Canadian Agency for Drugs and Technology in Health; CAR-T = chimeric antigen receptor t-cell therapy; FDA = Food and Drug Administration (US); HTA = health technology assessment; pCODR = pan-Canadian Oncology Drug Review; NICE = The National Institute for Health and Care Excellence (UK).

**Table 2:** Illustrative quotes for themes on sources of uncertainty identified in pCODR documents.

THEME (FREQUENCY)	QUOTATION
TRIAL VALIDITY (50%) SELECTION BIAS REPORTING BIAS PERFORMANCE BIAS ATTRITION BIAS	“Uncertainties about the heavily pre-treated patient population” “The open label nature of the trials might introduce the risk of reporting and performance biases, as the study participants and the investigators were aware of the treatment assignments.”
POPULATION (47%) ECOG	“From a methodological perspective, the low number of Canadian patients in the study make it uncertain how generalizable results are to the broader Canadian population.”
COMPARATORS (40%) NO COMPARATORS INAPPROPRIATE COMPARATORS	“Substantial uncertainty due to non-comparative data” “Uncertainty in results of indirect comparisons”
OUTCOMES (72%) UNVALIDATED ENDPOINTS MISSING DATA	“Progression-free survival may be a surrogate outcome for overall survival, but it has not been determined if benefits of PFS [progression-free disease] translates into overall survival benefits in patients with pancreatic neuroendocrine tumors.” “Modest improvement in progression-free survival” “There were uncertainties with regard to the magnitude of the progression-free survival benefit” “Neither study reported quality of life data”
INTERVENTION (83%) DURATION OF TREATMENT ADOPTION FEASIBILITIES BUDGET IMPACT	“pERC acknowledged a substantial uncertainty regarding duration of treatment” “pERC noted that the administration of intravenous daratumumab is resource-intensive due to the duration, frequency, and changing pattern of dosing” “[There is a] concern that implementation could lead to significantly increased resource utilization (e.g., nursing, pharmacy, clinic, and chemotherapy chair time”

## Data Availability

Data sharing is not applicable to this article to protect the identities of the participants. No new data was generated in the second phase of this study.

## References

[1] Love-Koh J., Peel A., Rejon-Parrilla J.C., Ennis K., Lovett R., Manca A., Chalkidou A., Wood H., Taylor M. (2018). The Future of Precision Medicine: Potential Impacts for Health Technology Assessment. PharmacoEconomics.

[2] Driedger S.M., Cooper E., Annable G., Brouwers M. (2018). ‘There is always a better way’: Managing uncertainty in decision making about new cancer drugs in Canada. Int. J. Health Plan. Manag..

[3] Booth C.M., Dranitsaris G., Gainford M.C., Berry S., Fralick M., Fralick J., Sue J., Clemons M. (2007). External influences and priority-setting for anti-cancer agents: A case study of media coverage in adjuvant trastuzumab for breast cancer. BMC Cancer.

[4] Lexchin J. (2018). Quality of evidence considered by Health Canada in granting full market authorisation to new drugs with a conditional approval: A retrospective cohort study. BMJ Open.

[5] Abola M.V., Prasad V. (2016). Characteristics and Conflicts of Public Speakers at Meetings of the Oncologic Drugs Advisory Committee to the US Food and Drug Administration. JAMA Intern. Med..

[6] Lexchin J. (2019). Financial conflicts of interest of clinicians making submissions to the pan-Canadian Oncology Drug Review: A descriptive study. BMJ Open.

[7] Ahn R., Herrera-Perez D., Prasad V. (2020). Characteristics of Public Comments Submitted to State Health Technology Assessment Programs in Oregon and Washington. JAMA Intern. Med..

[8] Peacock S.J., Regier D.A., Raymakers A.J.N., Chan K.K.W. (2019). Evidence, values, and funding decisions in Canadian cancer systems. Health Manag. Forum.

[9] Chafe R., Culyer A., Dobrow M., Coyte P.C., Swaka C., O’Reilly S. (2011). Access to Cancer Drugs in Canada: Looking beyond Coverage Decisions (Accès aux médicaments pour traiter le cancer au Canada: Voir au-delà des décisions concernant la couverture). Healthc. Policy.

[10] Andersen S.K., Penner N., Chambers A., Trudeau M.E., Chan K.K., Cheung M. (2019). Conditional Approval of Cancer Drugs in Canada: Accountability and Impact on Public Funding. Curr. Oncol..

[11] Vreman R.A., Naci H., Goettsch W.G., Mantel-Teeuwisse A.K., Schneeweiss S.G., Leufkens H.G.M., Kesselheim A.S. (2020). Decision Making under Uncertainty: Comparing Regulatory and Health Technology Assessment Reviews of Medicines in the United States and Europe. Clin. Pharmacol. Ther..

[12] Raymakers A.J.N., Regier D.A., Peacock S.J. (2020). Health-Related Quality of Life in Oncology Drug Reimbursement Submissions in Canada: A Review of Submissions to the pan-Canadian Oncology Drug Review. Cancer.

[13] Sandelowski M. (2000). Whatever Happened to Qualitative Description?. Res. Nurs. Health.

[14] Meyers D.E., Jenei K., Chisamore T.M., Gyawali B. (2021). Evaluation of the Clinical Benefit of Cancer Drugs Submitted for Reimbursement Recommendation Decisions in Canada. JAMA Intern. Med..

[15] Davis C., Naci H., Gurpinar E., Poplavska E., Pinto A., Aggarwal A. (2017). Availability of evidence of benefits on overall survival and quality of life of cancer drugs approved by European Medicines Agency: Retrospective cohort study of drug approvals 2009-13. BMJ.

[16] Raymakers A.J.N., Jenei K.M., Regier D.A., Burgess M.M., Peacock S.J. (2021). Early-Phase Clinical Trials and Reimbursement Submissions to the Pan-Canadian Oncology Drug Review. PharmacoEconomics.

[17] Srikanthan A., Penner N., Chan K., Sabharwal M., Grill A. (2018). Understanding the Reasons for Provincial Discordance in Cancer drug Funding—A Survey of Policymakers. Curr. Oncol..

[18] National Institute for Health and Clinical Excellence (2021). Cancer Drugs Fund (CDF) | Access to Treatment | Cancer Research UK. https://www.cancerresearchuk.org/about-cancer/cancer-in-general/treatment/access-to-treatment/cancer-drugs-fund-cdf.

[19] Bryan S., Mitton C., Donaldson C. (2014). Breaking the Addiction to Technology Adoption. Health Econ..

[20] Skedgel C., Younis T. (2016). The Politicization of Oncology Drug Funding Reviews in Canada. Curr. Oncol..

[21] Skedgel C., Wranik D., Hu M. (2018). The Relative Importance of Clinical, Economic, Patient Values and Feasibility Criteria in Cancer Drug Reimbursement in Canada: A Revealed Preferences Analysis of Recommendations of the Pan-Canadian Oncology Drug Review 2011–2017. PharmacoEconomics.

[22] Hilal T., Sonbol M., Prasad V. (2019). Analysis of Control Arm Quality in Randomized Clinical Trials Leading to Anticancer Drug Approval by the US Food and Drug Administration. JAMA Oncol..

[23] Naci H., Davis C., Savović J., Higgins J.P.T., Sterne J., Gyawali B., Romo-Sandoval X., Handley N., Booth C.M. (2019). Design characteristics, risk of bias, and reporting of randomised controlled trials supporting approvals of cancer drugs by European Medicines Agency, 2014-16: Cross sectional analysis. BMJ.

